# Numerical simulation and experiment of double chamber brake based on CFD

**DOI:** 10.1038/s41598-023-45010-9

**Published:** 2023-10-18

**Authors:** Liu Yuhao, Qu Pu, Li Qiang

**Affiliations:** School of mechanical and electrical engineering, North University of China, Taiyuan, 030051 China

**Keywords:** Energy science and technology, Engineering, Physics

## Abstract

The artillery firing process will instantly produce high-temperature and high-pressure gunpowder gas, this process will produce shock waves. The gunpowder gas has a limited effect on the projectile during the firing and ballistic after-effects period, however, it has a very obvious effect on the stability of the gun body, and the reduction of the stability of the gun body directly affects the firing accuracy and the safety of the firing personnel. Based on the method of Computational Fluid Dynamics (CFD), numerical simulation is carried out, and the structure and flow parameters of the muzzle flow field are obtained by using three-dimensional Euler's control equation, gas equation of state, and k-epsilon model, as well as dynamic mesh technology. By comparing the flow parameters of the brake before and after optimization, and analyzing the results obtained from the 8-round firing experiments, the efficiency of the optimized brake is increased by 8.2%, and the deviation between the experimental data and the simulation results is only 10.5%, which not only verifies the accuracy of the numerical simulation calculations but also verifies the optimized brake's good retracting effect. The results of the study can provide a reference for the optimization and design of the double-chamber brake.

## Introduction

Generally speaking, the role of the muzzle brake has two main points one is to reduce the recoil kinetic energy. When the recoil part of the mass and recoil length is certain, it can reduce the force on the gun frame when firing, thus reducing the longitudinal size of the gun frame and reducing the weight of the gun; or when the recoil resistance is certain, then shorten the recoil length. The second is to use a uniform gun mount. To install the gun body with different power on the same gun mount, from the mechanical point of view, it is only necessary to ensure that the free recoil kinetic energy is equal.

However, the aftereffects of the gunpowder gas on the gun body during the after-effects period^1–5^ are obvious, and the recoil impulse can reach more than 20% of the overall recoil impulse of the gun. By distributing the flow of gunpowder gas in the after-effect period^6–10^ hanging the direction of air velocity to produce an overall forward impulse, this impulse can provide a recoil force to the gun body and reduce the recoil impulse generated by the corresponding combined force of the gun bore to reduce the firing load to achieve the recoil effect, which effectively improves the firing accuracy^11–18^.

Due to the complexity of the structure of the gun itself and the difficulty in mastering hydrodynamics, as well as the large amount of human and material resources required to perform weapon testing in the near-flow field and far-flow length, the validation cost is very high. There are very few calculations and simulations on double-chambered brakes and fewer experiments^19–21^ to reveal the flow field changes caused by the muzzle retractor. In the last decade or so, computational fluid dynamics (hereafter referred to as "CFD")^22–28^ has developed greatly, replacing some approximate computational and graphical methods in classical fluid dynamics. All problems involving fluid flow^29,30^, heat exchange, molecular transport^31^, and other phenomena can be analyzed and simulated almost by means of computational fluid dynamics. CFD is being used to solve a lot of difficult real-world problems^32–34^, and is playing an important role as a research tool in aeronautics, nano-materials^35,36^, national defense, etc.^37,38^.

Using the software Ansys Fluent in Computational Fluid Dynamics, with the help of Ansys Fluent, you can create advanced physical models and analyze a variety of fluid phenomena so that all the work is done in the same customizable intuitive space. Currently, complex simulation tasks such as downscaling, multiphase flow, fluid–solid coupling, etc. are accomplished in this software, which has been widely recognized.

In this paper, taking the double chamber brake as the research object, numerical simulation of artillery firing with or without muzzle brake is carried out by establishing a mathematical-physical model based on the method of fluid computational mechanics. According to the simulation results to optimize the design, and finally,the optimized numerical simulation results are compared with the experimental results to verify the accurate availability of the numerical simulation simulation calculation.

## Methods

### Modeling

**Figure 1:**
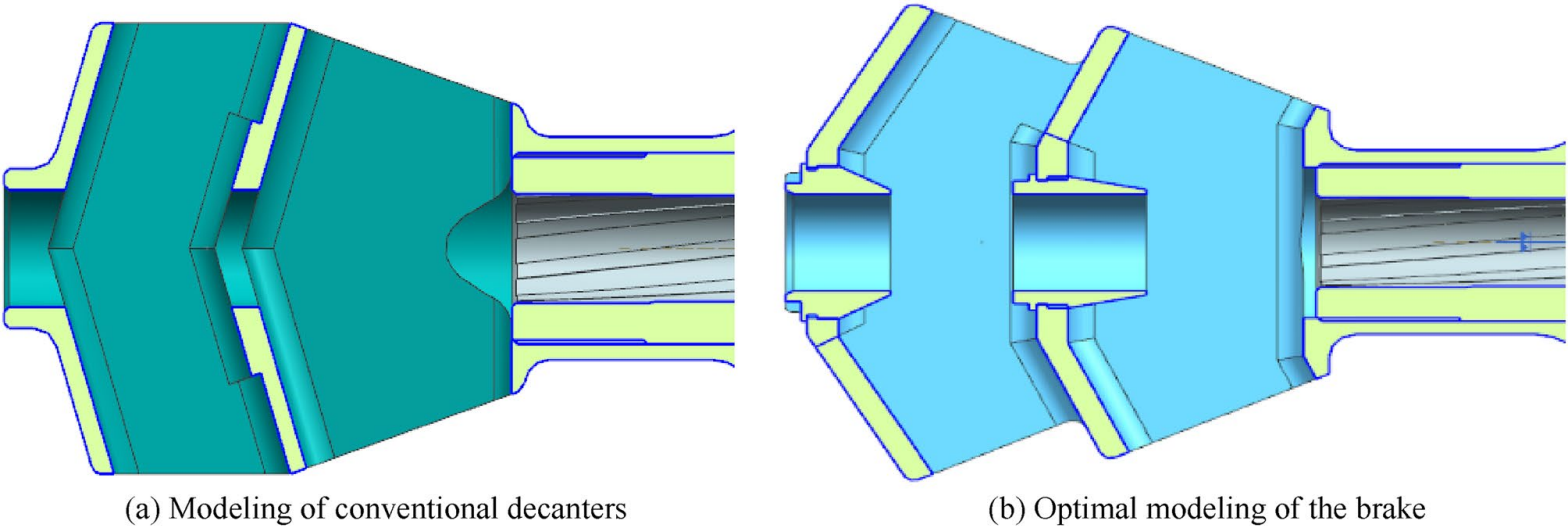
Modeling of the brake.

Figure 1a shows the conventional brake model. The central single hole of the baffle scheme is 22 mm in diameter, with 2 chambers and 2 rows of side apertures, and the wall of the side aperture is at an angle of 72° to the bore axis. The width of the baffle is 85 mm, the height is 60 mm, and the total length is 166 mm.

As shown in Fig. 1b, the model of the optimized brake is shown. The diameter of the central bullet hole is still 22 mm, 2 recoil chambers are retained, 2 rows of side apertures, and the angle between the side apertures and the bore axis is changed to 60°. The maximum height of the brake is 60 mm, the width is set to 90 mm, the height of the brake cavity is 52 mm, and the total length is extended to 180 mm.

The adjustment of the angle between the side aperture and the bore axis of the optimized type of brake makes the expanded powder gas more efficiently discharged to the side aperture, reduces the backward axial reaction force and further increases the forward lateral reaction force, which is a reference value for the study of the improvement of the efficiency of the brake.

**Figure 2:**
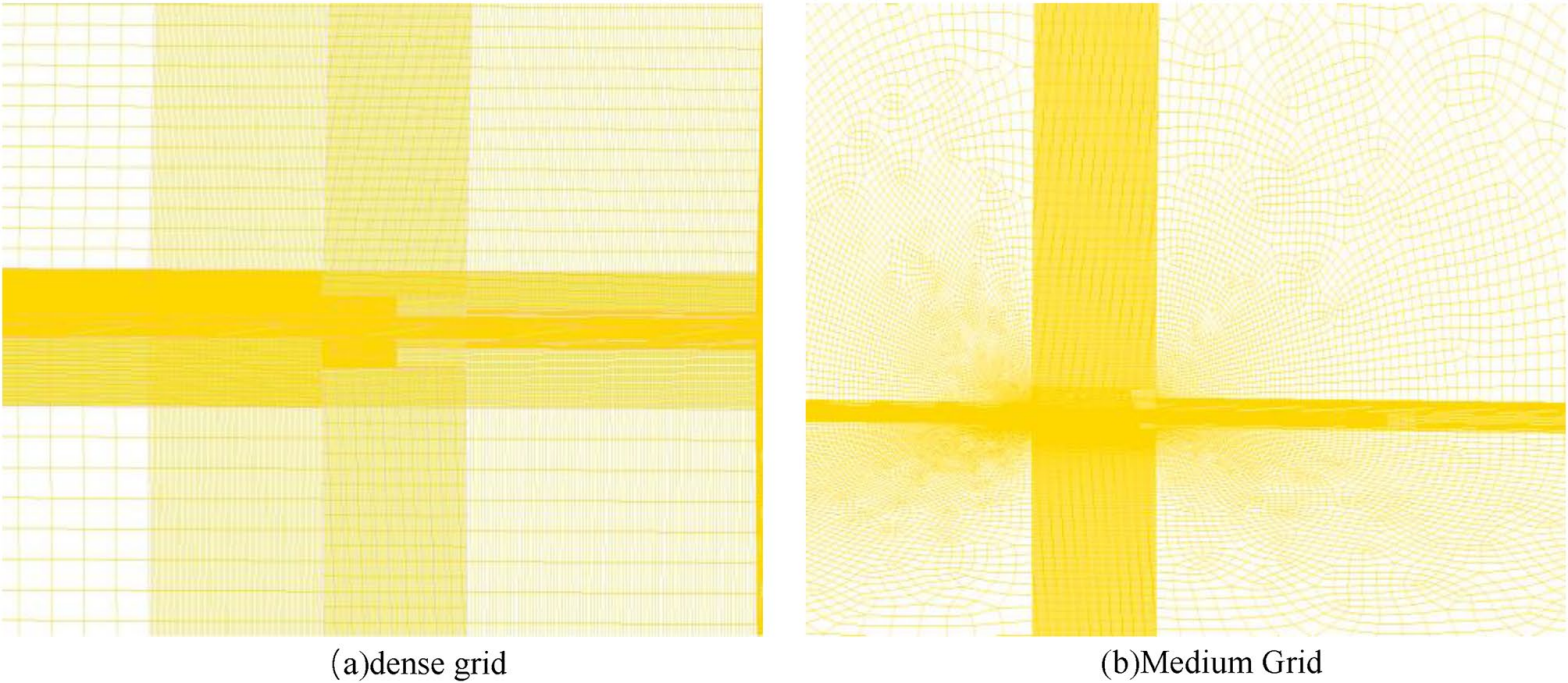
Grid-independent verification model.

A model of the computational grid area is shown in Fig. 2a,b. The gun muzzle flow field is external to the atmospheric environment and has no actual boundary. For such problems, it can be considered that a suitable external spatial region is selected as the study area. In the process of artillery firing, the high-pressure and violently burning gunpowder gas is ejected from the muzzle section and expands violently, interacting with the atmosphere to form a complex gas jet flow field. The flow field will show the complex structure of the excitation system such as reflection intersection and Mach disk, and the area of the flow field will become larger and larger as time goes by. Here, a quarter cylinder with a radius of 800 mm and a length of 4000 mm is selected as the external calculation area.

**Figure 3:**
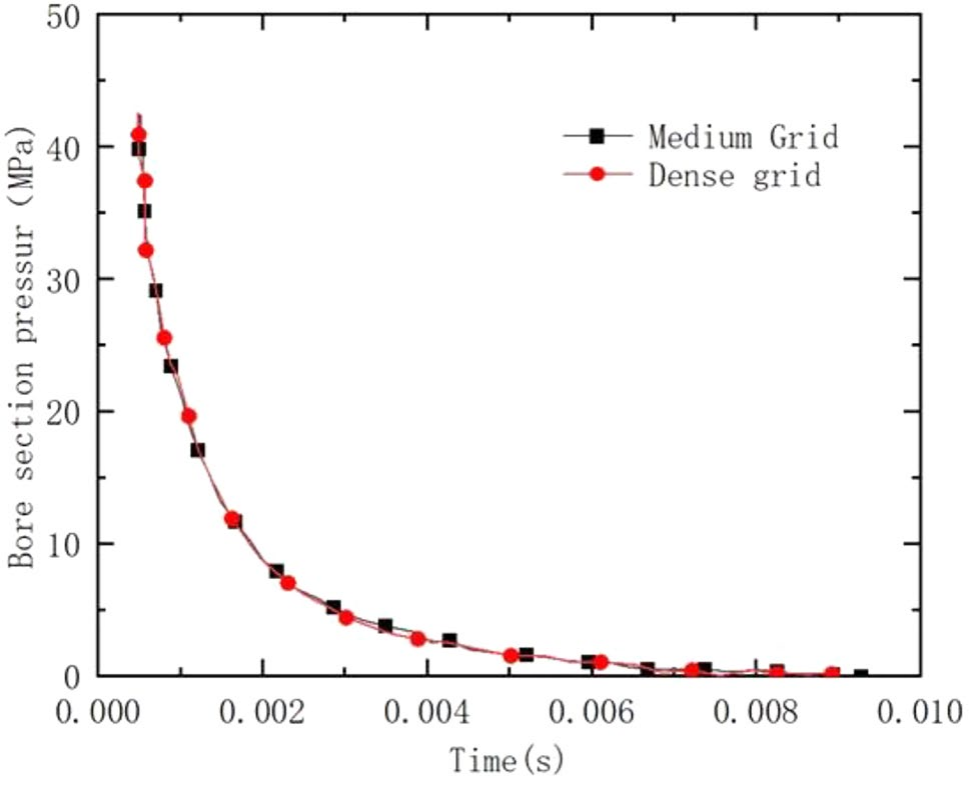
Comparison of pressure at the bore section in the case of dense grid.

As in Fig. 3, the medium and dense grids are drawn with structural grids. The grid aspect ratio is close to 0.97, and the maximum deviation angle of the opposite side is no more than 30°. The number of medium grids is 886,700 and the number of dense grids is 1,665,300. In the numerical simulation, except for the change of grid density, all the parameters are kept constant, and the pressure of the bore section is monitored, and the results are shown in Fig. 3. The medium grid can be used, which has a positive impact on improving the solution efficiency.

### Theoretical analysis

In order to simplify the problem of airflow in the gun chamber during the after-effect period, the following assumptions are introduced.

1. The flow is quasi-constant.
2. The flow is one-dimensional, even if the cross-sectional area of the flow varies, the radial fractional velocity of the flow and the effect of the inhomogeneity of the flow parameters in the cross-section are corrected by the corresponding coefficients.
3. The flow is isentropic, i.e., the heat exchange between the airflow and the chamber wall is neglected, and the friction caused by the viscosity of the gas is neglected. The resulting errors are also corrected by the coefficients.
4. The gunpowder gas is regarded as a complete gas (or ideal gas) and the mass force is neglected.

Under the above assumptions, the three-dimensional constant isentropic flow theory in gas dynamics can be applied to study the flow-void problem of gunpowder gas in the post-effect period.

#### Control equations

According to the above assumptions, the Euler equation for three-dimensional non-constant compressible flow in the gun bore can be established under the premise of continuous medium mechanics.

1 $$\frac{\partial U}{{\partial t}} + \frac{{\partial F^{x} (U)}}{\partial x} + \frac{{\partial F^{y} (U)}}{\partial x} + \frac{{\partial F^{z} (U)}}{\partial x} = 0$$

Among them:

$$U = \left[ \begin{gathered} \rho \hfill \\ \rho u \hfill \\ \rho v \hfill \\ \rho w \hfill \\ E \hfill \\ \end{gathered} \right];\quad F^{x} (U) = \left[ \begin{gathered} \rho u \hfill \\ \rho u^{2} + p \hfill \\ \rho uv \hfill \\ \rho uw \hfill \\ u(E + p) \hfill \\ \end{gathered} \right];\quad F^{y} (U) = \left[ \begin{gathered} \rho u \hfill \\ \upsilon uv \hfill \\ \rho v^{2} + p \hfill \\ \rho uw \hfill \\ v(E + p) \hfill \\ \end{gathered} \right];\quad F^{z} (U) = \left[ \begin{gathered} \rho w \hfill \\ \rho uw \hfill \\ \rho vw \hfill \\ \rho w \hfill \\ w(E + p) \hfill \\ \end{gathered} \right].$$

The equation of state and auxiliary equations are:

2 $$p = (\gamma - 1)\left[ {E - \frac{1}{2}\rho (u^{2} + v^{2} + w^{2} )} \right]$$

3 $$h = \frac{E + p}{\rho } = \frac{\gamma }{(\gamma - 1)}\frac{p}{\rho } + \frac{1}{2}(u^{2} + v^{2} + w^{2} )$$

4 $$C^{2} = (\gamma - 1)\left[ {h - \frac{1}{2}(u^{2} + v^{2} + w^{2} )} \right]$$

where *ρ* is the gas density; *E* is the total energy of the gas; *u*, *v*, *w* is the velocity component of the gas along the *x*, *y*, *z* direction, respectively; *p* is the gas flow pressure; *γ* is the specific heat ratio; *γ* is the enthalpy of the gas flow; *c* is the local speed of sound.

In this paper, the coordinate system is established with the projectile launch direction as the positive direction of the coordinate axis and the moment of projectile movement to the rifling port as the initial time, i.e., T = 0.

Gas pressure calculation mainly requires solving five unknown quantities of pressure, temperature, density,velocity of the piston and displacement, here we establish a mathematical model by gas motion and piston motion for solving. In this paper, we take time T as the independent variable and the dependent variable as the previous five unknown quantities and establish the equation for the joint solution. The expressions are as follows:

1. The equation of the variation law of gas density inside the gas conduction chamber:
  5 $$\frac{{{\text{d}}\rho_{{\text{q}}} }}{{{\text{dt}}}} = \frac{{{\text{q}}_{{{\text{mb}}}} - {\text{q}}_{{{\text{mq}}}} - \rho_{{\text{q}}} {\text{s}}_{{\text{n}}} {\text{v}}_{{\text{h}}} }}{{{\text{v}}_{{{\text{q}}0}} + {\text{s}}_{{\text{h}}} {\text{x}}_{{\text{h}}} }}$$
  in the formula:
  $$\begin{aligned} {\text{q}}_{{{\text{mq}}}} = \mu_{{\text{q}}} \Delta {\text{s}}_{{\text{h}}} \sqrt {\lambda \left( {\frac{2}{\gamma + 1}} \right)^{{\frac{\gamma + 1}{{\gamma - 1}}}} {\text{p}}_{{\text{q}}} \rho_{{\text{q}}} } \\ & \quad \quad q_{mb} \left\{ {\begin{array}{*{20}l} {\mu_{b} S_{b} \sqrt {\gamma \left( {\frac{2}{\gamma + 1}} \right)^{{\frac{\gamma + 1}{{\gamma - 1}}}} p_{p} \rho_{p} } } \hfill & {{\text{Positive}}\,{\text{critical}}\,{\text{Flow:}}\,{\text{P}}_{q} \le \zeta p_{p} ;} \hfill \\ {\mu_{b} S_{b} \sqrt {\frac{2\gamma }{{\gamma - 1}}p_{p} \rho_{p} \left[ {\left( {\frac{{p_{q} }}{{P_{p} }}} \right)^{{\frac{2}{\gamma }}} - \left( {\frac{{p_{q} }}{{p_{p} }}} \right)^{{\frac{\gamma + 1}{\gamma }}} } \right]} } \hfill & {{\text{Reverse}}\,{\text{subcritical}}\,{\text{flow:}}\,{\text{P}}_{p} \zeta < {\text{P}}_{q} < p_{p} ;} \hfill \\ { - \mu_{b} S_{b} \sqrt {\frac{2\gamma }{{\gamma - 1}}p_{q} \rho_{q} \left[ {\left( {\frac{{p_{p} }}{{p_{q} }}} \right)^{{\frac{2}{\gamma }}} - \left( {\frac{{p_{p} }}{{p_{q} }}} \right)^{{\frac{\gamma + 1}{\gamma }}} } \right]} } \hfill & {{\text{Reverse}}\,{\text{subcritical}}\,{\text{flow:}}\,{\text{P}}_{q} \zeta < p_{p} < p_{q} ;} \hfill \\ { - \mu_{b} S_{b} \sqrt {\gamma \left( {\frac{2}{\gamma + 1}} \right)^{{\frac{\gamma + 1}{{\gamma - 1}}}} p_{q} \rho_{q} } } \hfill & {{\text{Reverse}}\,{\text{critical}}\,{\text{flow:}}\,{\text{P}}_{p} \le {\text{P}}_{q} \zeta_{ \circ } } \hfill \\ \end{array} } \right. \\ \xi & = \left( {\frac{2}{\gamma + 1}} \right)^{{\frac{\gamma }{\gamma - 1}}} \\ \end{aligned}$$
2. Pressure change equation of gas chamber:
  6 $$\frac{{{\text{d}}\rho_{{\text{q}}} }}{{{\text{dt}}}} = \frac{\gamma - 1}{{V_{{{\text{q}}0}} + S_{{\text{h}}} X_{{\text{h}}} }}\left( { - \frac{{{\text{d}}Q_{{\text{r}}} }}{{{\text{dt}}}} + {\text{e}}_{{\text{i}}} {\text{q}}_{{{\text{mb}}}} - {\text{e}}_{{\text{q}}} {\text{q}}_{{{\text{mq}}}} - \frac{{\gamma {\text{p}}_{{\text{q}}} v_{k} S_{k} }}{\gamma - 1}} \right)$$
3. Equation of state:
  7 $${\text{p}}_{{\text{q}}} = \rho_{{\text{q}}} RT_{{\text{q}}}$$
  The meaning of each symbol in the formula is:
  $$\begin{aligned} & \mu_{{\text{b}}} - Flow\,coefficient \\ & \xi - Critical\,pressure \\ & \Delta S_{{\text{h}}} - Clearance\,area\,between\,piston\,and\,gas\,chamber \\ & V_{{{\text{q}}0}} - Initial\,volume\,of\,the\,gas\,conductivity\,chamber \\ & S_{{\text{h}}} - Piston\,cross{ - }sectional\,area \\ & P_{{\text{p}}} - Pressure\,of\,inflowing\,gas\,into\,the\,pilot\,chamber \\ & \rho_{{\text{p}}} - Density\,of\,the\,inflow\,gas\,into\,the\,conductive\,chamber \\ & T_{{\text{p}}} - Temperature\,of\,the\,inflow\,gas\,into\,the\,conductive\,chamber \\ & \frac{{{\text{d}}Q}}{{{\text{dt}}}} - Pressure\,of\,the\,gas\,in\,the\,gas\,conductor\,chamber \\ & R - Gas\,constants \\ & \rho_{{\text{q}}} - Gas\,density\,inside\,the\,gas\,conductivity\,chamber \\ & T_{{\text{q}}} - Gas\,density\,inside\,the\,gas\,conductivity\,chamber \\ & F_{{\text{q}}} - Air\,chamber\,surface\,area \\ & F_{{{\text{q}}0}} - Initial\,surface\,area\,of\,air\,chamber \\ & {\text{d}}_{{\text{h}}} - Piston\,diameter \\ & R_{{\text{f}}} - Resistance\,to\,piston \\ & {\text{m}}_{{\text{h}}} - The\,sum\,of\,the\,masses\,of\,the\,moving\,parts\,with\,the\,piston\,and\,the\,heat\,loss \\ \end{aligned}$$

Under the assumption of continuous medium mechanics, airflow can be described by the three-dimensional Euler equation with the gas equation of state. In calculating flow under hypersonic conditions, the Reynolds number is so high that the viscosity of the gas can usually be neglected. The flow of fluids is governed by physical conservation laws, and the above control equations are mathematical descriptions of these conservation laws. Computational mechanics of fluids is based on the above control equations to accurately calculate the flow of fluids.

### Initial conditions

This simulated gun uses a single charge, and the set of ballistic equations within the single charge can be summarized as follows:

1. Shape function:
  8 $$\psi = xZ(1 + \lambda Z + \mu Z^{2} )$$
2. Combustion velocity equation:
  9 $$\frac{dZ}{{dt}} = \frac{{u_{1} p^{n} }}{{e_{1} }}$$
3. Projectile equation of motion:
  Equation of motion expressed in terms of average pressure and secondary work factor *ψ*:
  10 $$\psi m = \frac{dv}{{dt}} = Sp$$
4. Basic equations of internal ballistics:
  11 $$Sp(l_{\psi } + l) = f\omega \psi - \frac{\theta }{2}\varphi mv^{2}$$
  in the formula:
  $$\begin{aligned} l_{\psi } & = l_{0} \left[ {1 - \frac{\Delta }{{\rho_{p} }}(1 - \psi ) - \alpha \Delta \psi } \right] \\ \theta & = k - 1 \\ \end{aligned}$$

**Figure 4:**
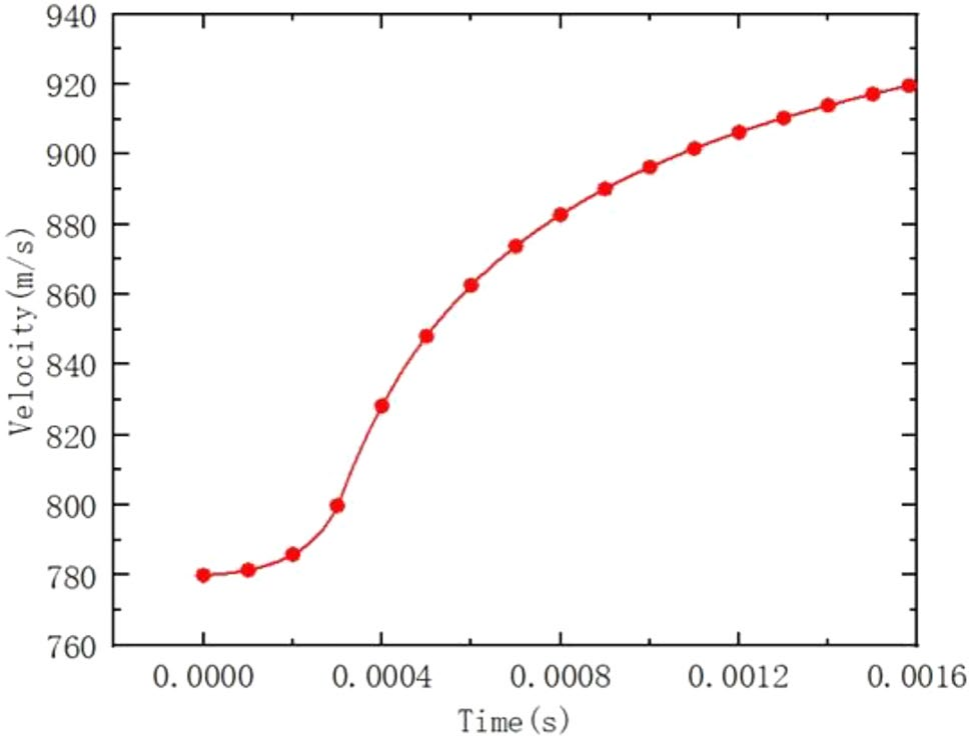
Time-speed curve.

**Figure 5:**
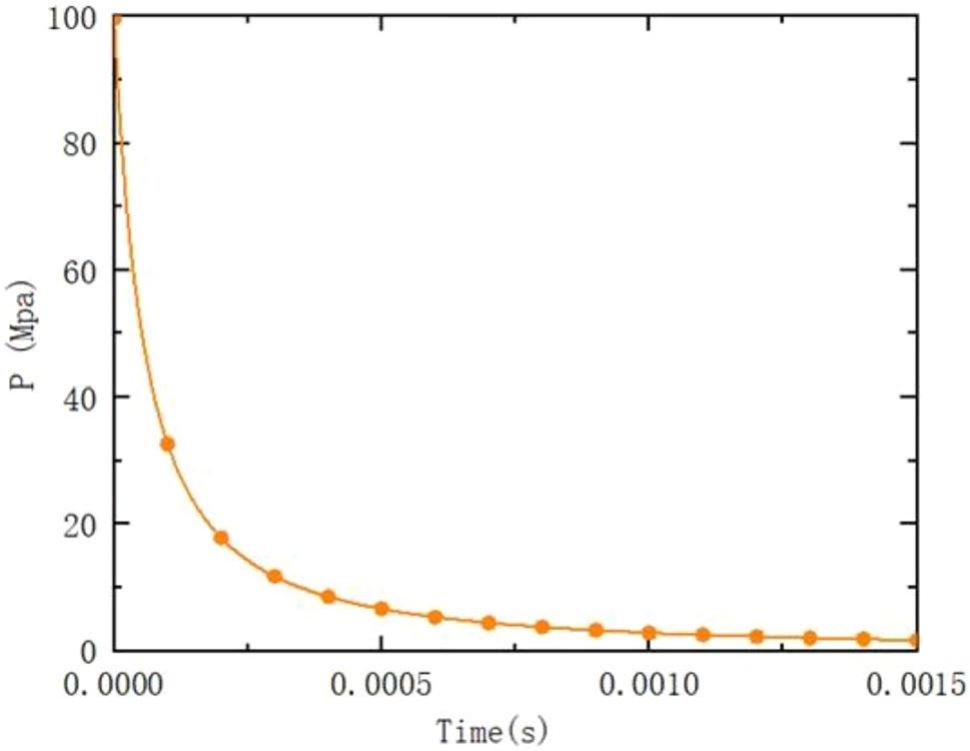
Time-pressure curve.

In this paper, the rifling end face is set as the entrance boundary, and the initial conditions and flow parameters of the powder gas are obtained by solving the internal ballistic program. The projectile trajectory equations are given by the UDF preparation. The exit boundary is set as a pressure exit and the rest is given as a solid wall boundary. The internal ballistic time velocity curve and time rifling pressure curve are shown in Figs. 4 and 5.

**Figure 6:**
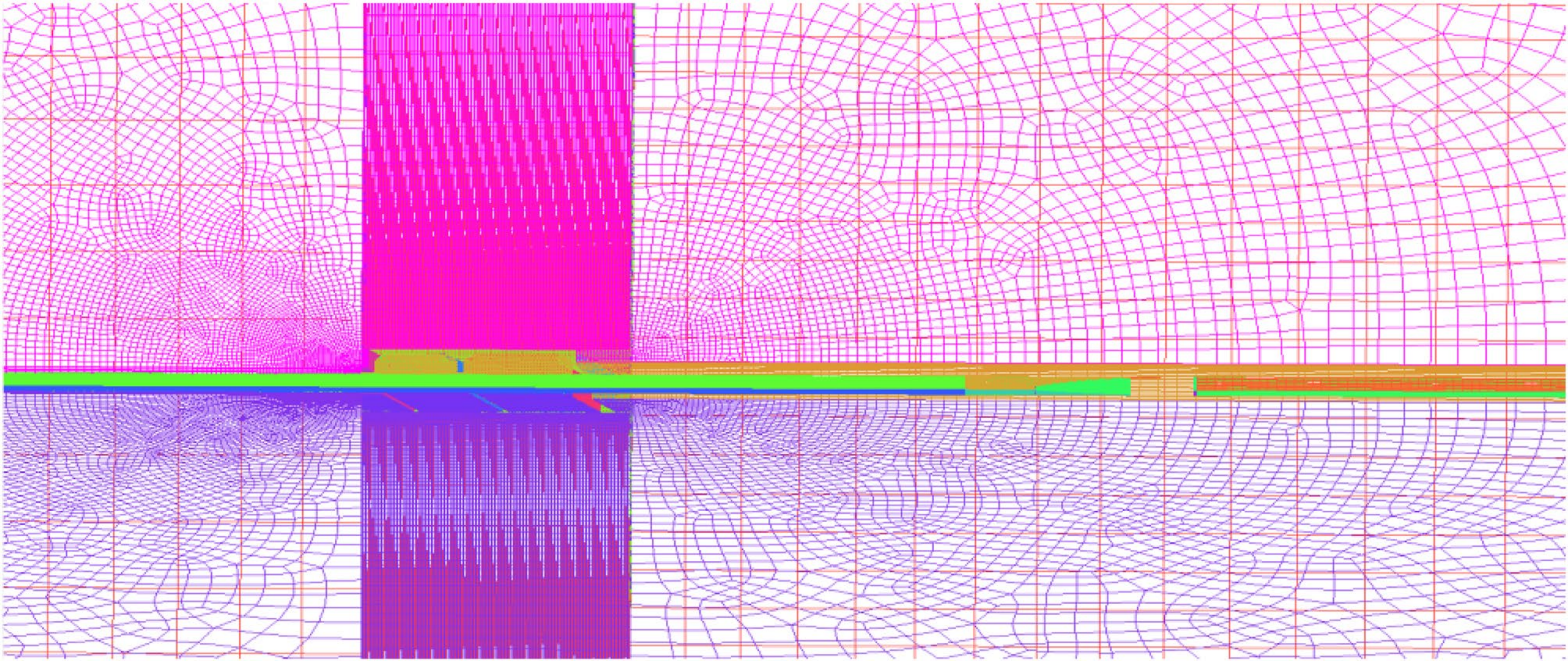
Computational domain grid model.

**Figure 7:**
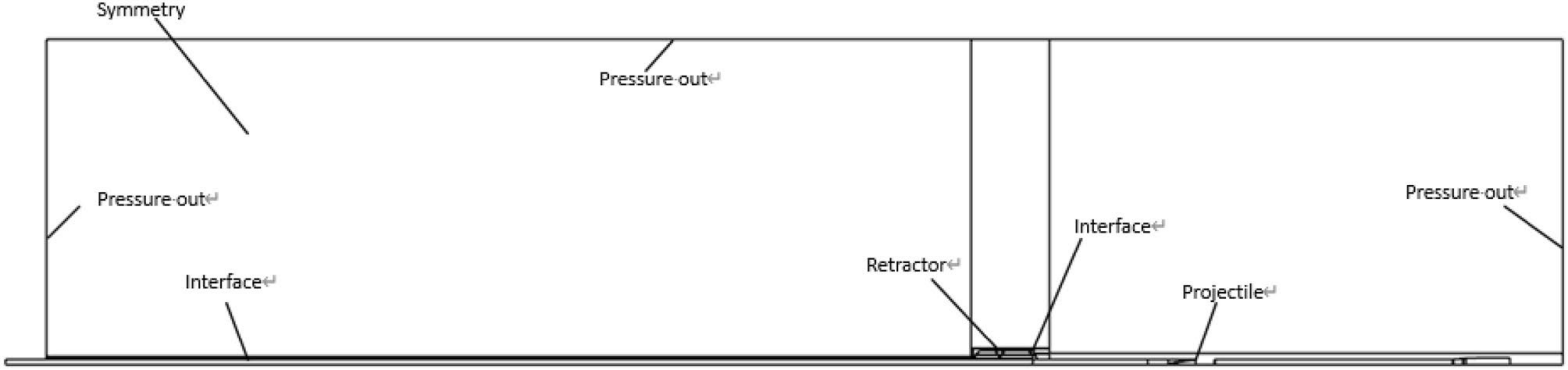
Initialized flow field.

Combining the results of the internal ballistic calculation above and the model of the computational grid area in Fig. 6 (where the schematic diagram can be obtained according to Fig. 7), the modal simulation initial conditions and the experimental initial conditions are plotted in a Table 1.

**Table 1:** Initial input conditions.

Discharge port.speed (m/s)	Starting pressure (MPa)	Bore temperature (K)	Thermal insulation coefficient	Bore gas	Loading quantity (kg)
780.50	99.64	2235.00	1.25	Ideal gas	0.032

## Numerical simulation

### Numerical simulation of the breechless brake

Numerical calculations based on the computational fluid dynamics method are carried out to obtain the bore flow parameters and the bore side aperture and central bullet aperture airflow parameters based on the computational fluid dynamics method to calculate the bore efficiency in the after-effect period of different schemes.

**Figure 8:**
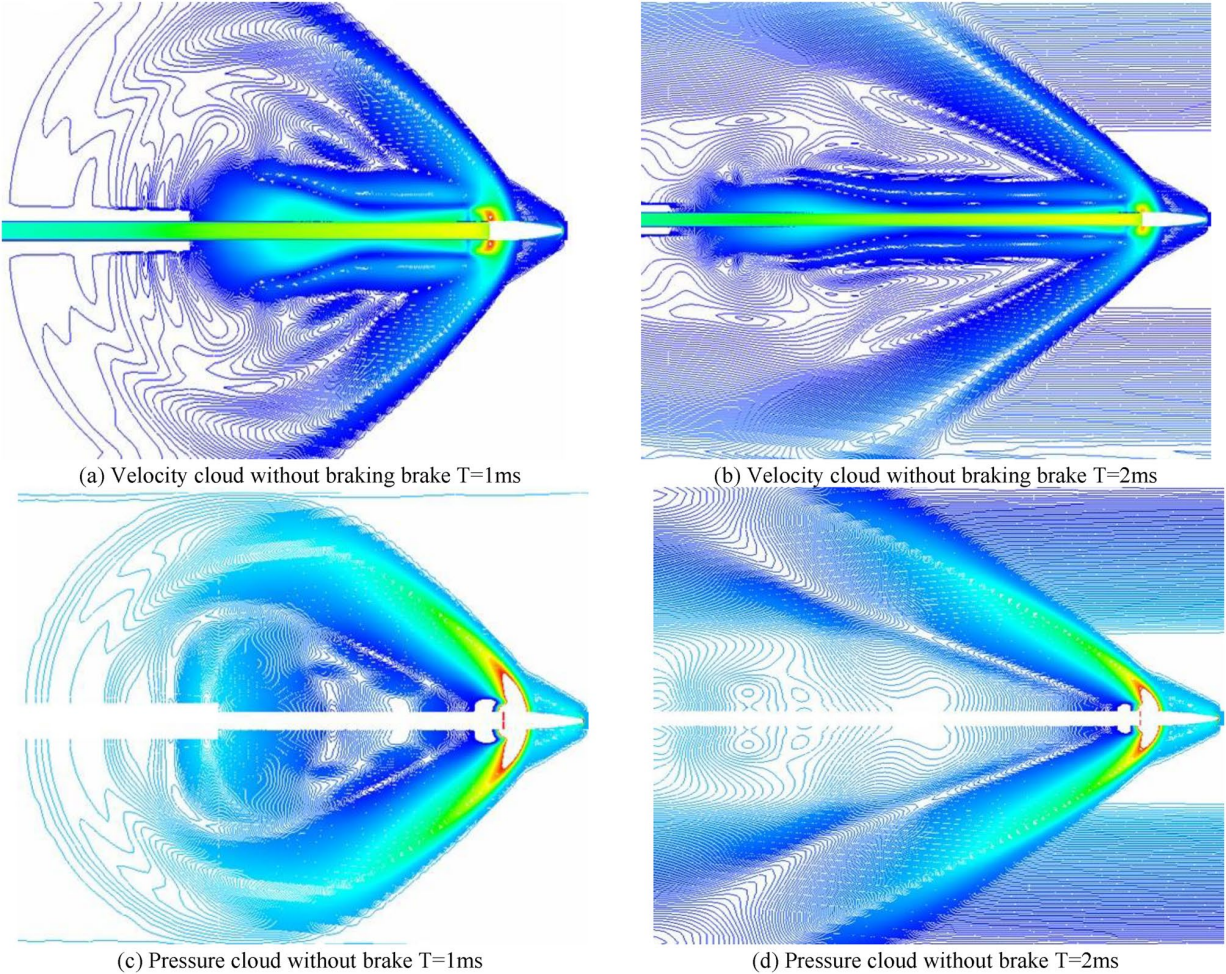
Pressure field of gas flow velocity at the orifice without brake.

As shown in Fig. 8, the velocity and pressure fields of the rifled gas flow without the brake are shown. When the high temperature and high-pressure gunpowder gas in the chamber is ejected from the chamber, it expands sharply outside the chamber, forming the chamber gas flow field with a complex surge system. Due to the large pressure difference inside and outside the nozzle, the high-pressure gas in the chamber expands sharply after entering the atmosphere after exiting the nozzle, and the pressure decreases and the velocity increases, forming a low-pressure supersonic free expansion zone in the area near the nozzle. The gas compresses the surrounding air during the expansion process, forming a positive excitation wave directly in front of the free expansion zone and an oblique excitation wave on the side. The high-speed low-pressure airflow in the free expansion zone increases in pressure and decreases in speed after the positive excitation wave is formed in front of the positive excitation wave and then forms a subsonic region. As the amount of gas in the launch tube becomes less and less, the pressure becomes lower and lower, the gas flow field gradually turns from expanding to shrinking until it gradually disappears.

### Numerical simulation of conventional two-cavity brake

**Figure 9:**
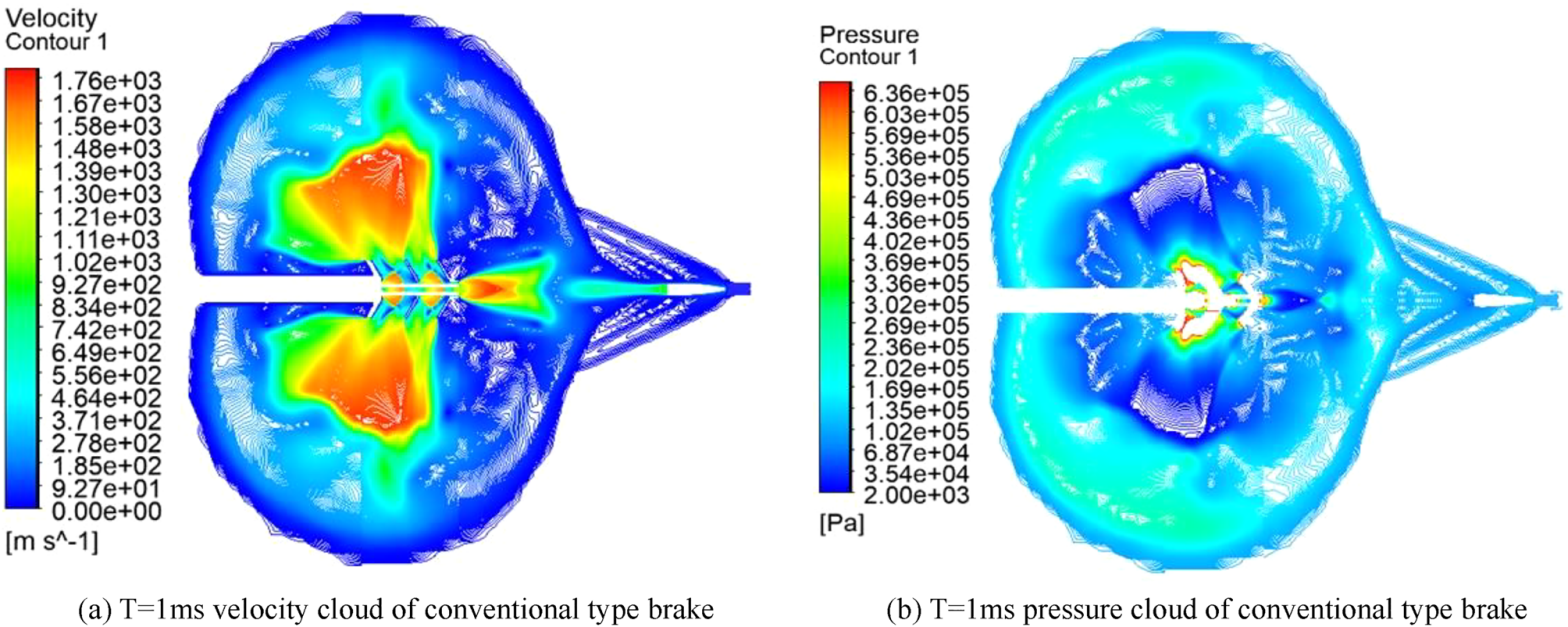
Velocity pressure cloud at different moments.

Figure 9 shows the velocity field and pressure field of the gas flow at the rifling of the conventional type brake before improvement. When the high-temperature and high-pressure gunpowder gas produced by the intense combustion in the chamber is instantaneously ejected through the chamber and enters the receding cavity in front, it expands rapidly in the receding cavity and forms a surge, and the gas is ejected from the first side aperture after passing through the surge. Due to the large pressure difference between the inside and outside of the side aperture, the high-pressure gas in the recession chamber is ejected through the side aperture and continues to expand sharply after entering the atmospheric environment, and the gas flow pressure decreases and the velocity increases, forming a flow field containing a complex surge system in the area near the side aperture. As the projectile moves forward in the brake, the high-pressure gas inside the brake is ejected from each side aperture in turn, forming a complex flow field near each side aperture, and as the gas continues to expand, the flow fields near each side aperture are coupled with each other, and the excitation systems in the flow field intersect and reflect, gradually forming a more complex flow field. In the process of gas ejection from the side aperture, the force acting on the front wall surface of the side aperture is much larger than the force acting on the rear wall surface, and the combined force of the walls of the brake in the after-effect period is forward to play the role of rejection. When the projectile flies out from the front of the brake, part of the gas in the bore is ejected from the central orifice in front of the brake, and because part of the gas has flowed out from the side orifice, the gas flow in the central orifice is greatly reduced.

### Numerical simulation of improved double-cavity brake

**Figure 10:**
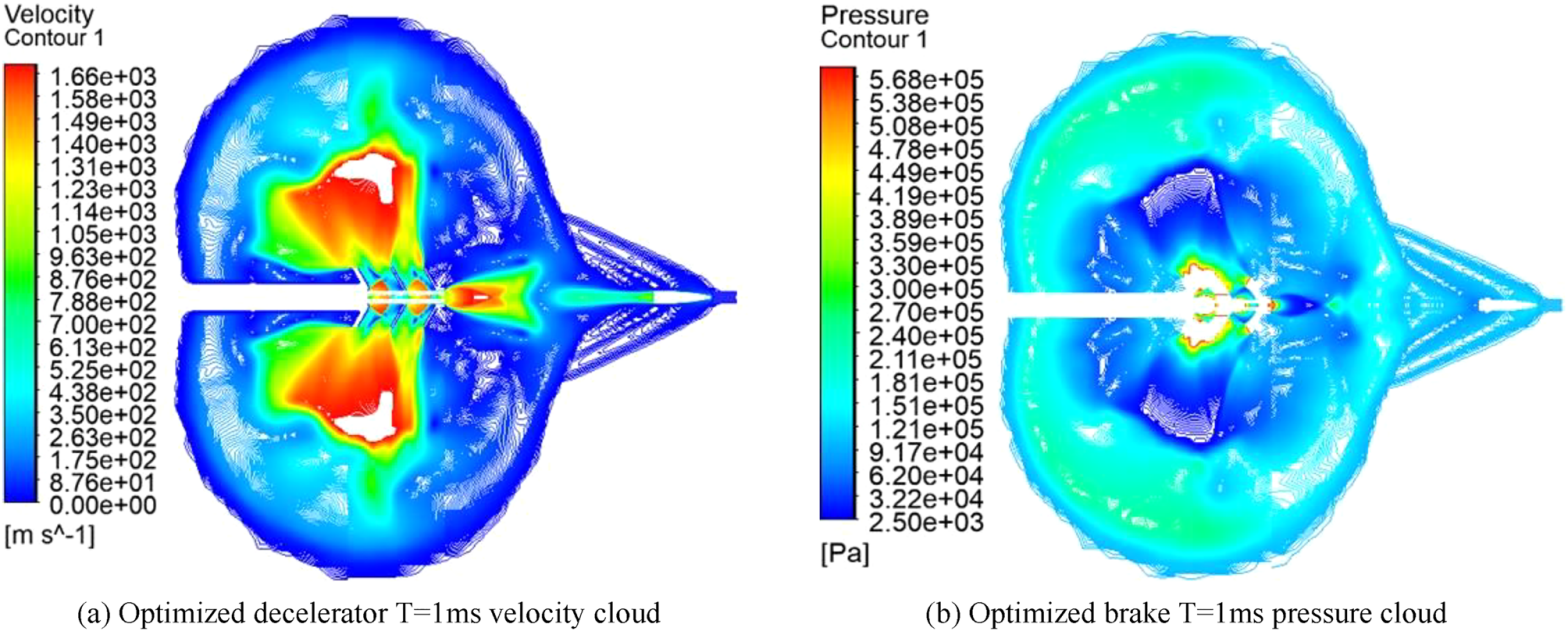
Optimized brake T = 1 velocity pressure cloud.

Figure 10 shows the velocity and pressure clouds for 1 ms after the numerical simulation of the modified double-cavity brake. The principle is the same as described above. The combined force of the walls of the regenerator is forward, which acts as the regenerative efficiency. A large amount of expansion gas is ejected from the side orifices, so the final gas flow from the central bullet hole is substantially reduced.

**Figure 11:**
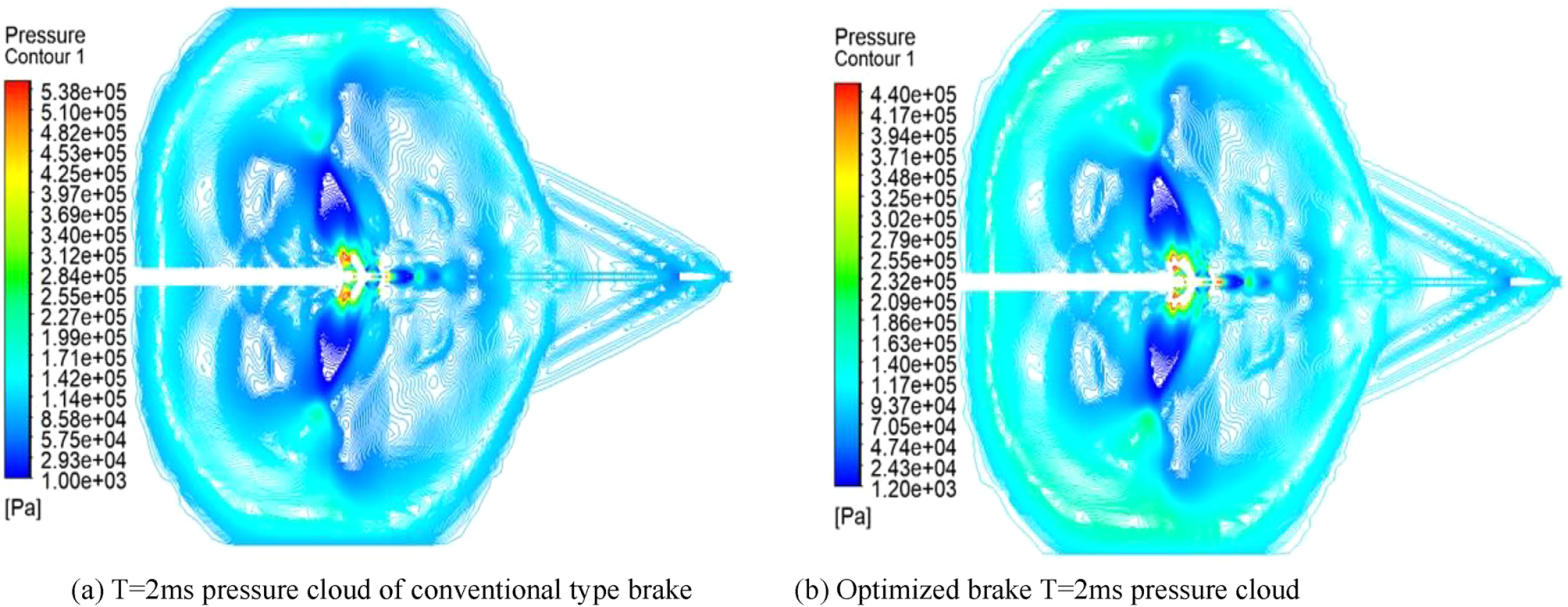
Comparison of T = 2 ms pressure clouds before and after optimization.

As shown in Fig. 11, the pressure cloud diagram of the 2 ms moment comparison between the traditional and optimized brake, the pressure at the extreme point decreases by 9.8e^4 Pa, the reduction rate reaches 18%, the airflow from the central bullet hole decreases significantly, the airflow pressure decreases significantly, the optimization of the double-chamber brake plays an effect.

**Figure 12:**
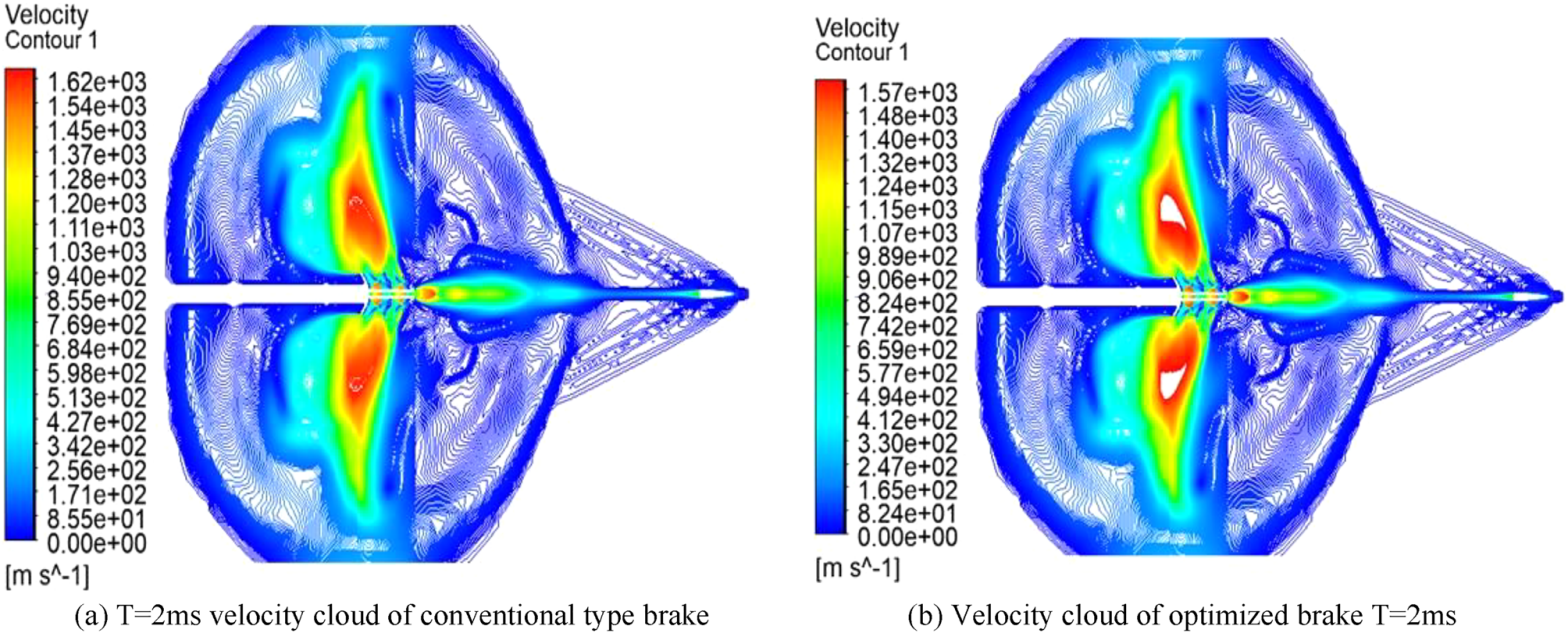
Comparison of T = 2 ms speed clouds before and after optimization.

As shown in Fig. 12, the velocity cloud diagram of the 2 ms moment comparison between the conventional and the optimized brake, it is obvious that the great value of velocity decreases from 1620 to 1570 at the 2 ms moment, and the reduction rate is 3%, and the velocity of middle airflow is at the great value point, and the distribution range shrinks inward.

### Comparative analysis with experiments

**Figure 13:**
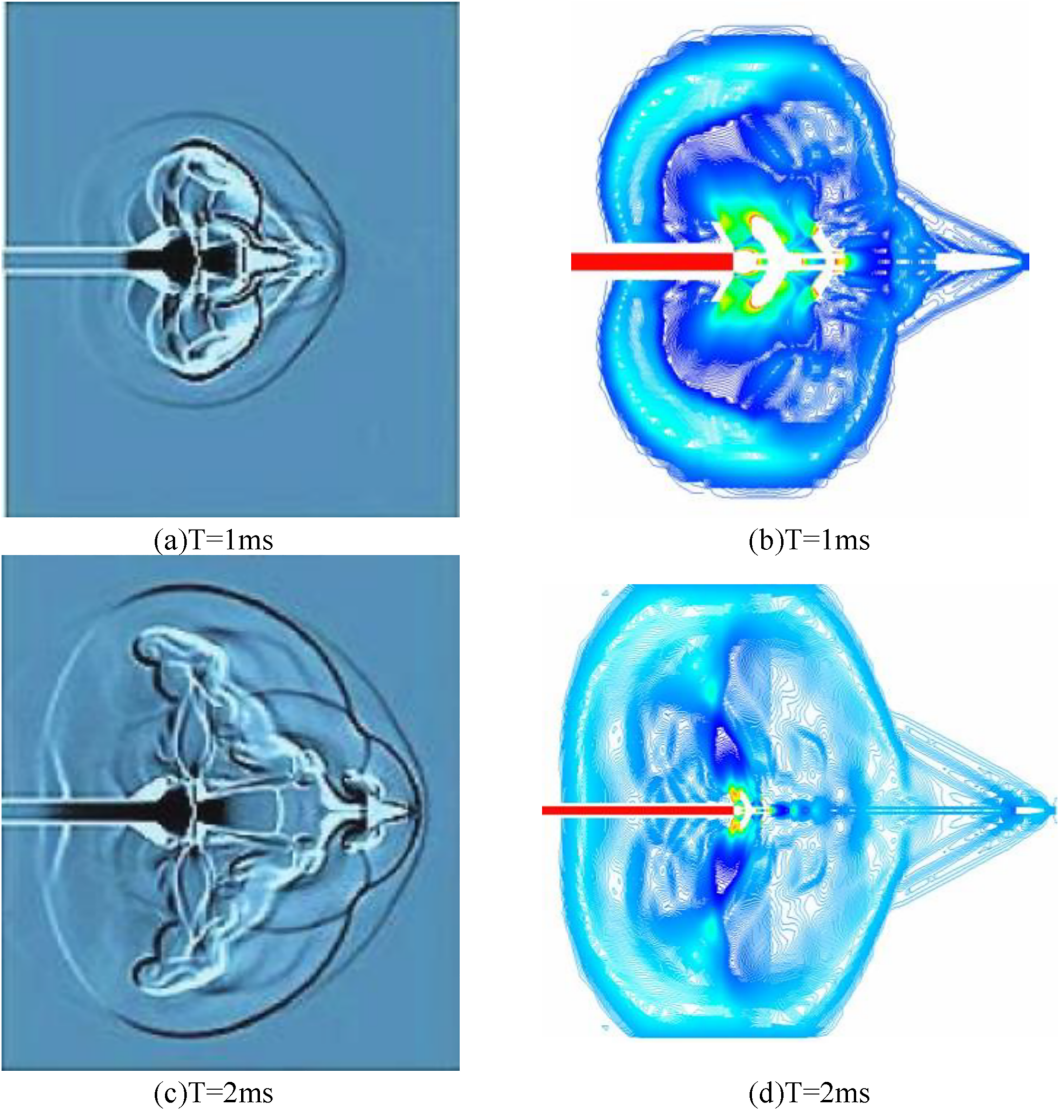
Rifled shock wave and experimental comparison cloud.

As shown in Fig. 13a,b, the comparison of the bore-sighted shock wave and the experimental T = 1 ms pre-bullet excitation field is shown. Since the external environment is still air and the internal airflow is only driven by a single initial jet, the wavefront surface of the shock wave is always spherical. The accuracy of the numerical simulation is demonstrated by the figure comparing the point explosion shock wavefront surface.

As shown in Fig. 13c,d for the borehole shock wave with the experimental T = 2 ms pressure farther field comparison. After the interaction between the rifled shock wave and the jet, the shock wave propagates to the farther side of the rifling. At this point, the restraining effect of the rifled shock wave on the jet is greatly weakened, and the supersonic jet acquires full development and enters a stage from relative stability to continuous decay; the projectile penetrates out of the Mach disk into the coronal air mass region, which has no stalling effect on the jet. The airflow after the rifled shock wave has stopped due to the decreasing pressure at the rifled mouth and the energy replenishment, and the rifled shock wave starts the free decay phase relying on its own energy to expand outward.

**Figure 14:**
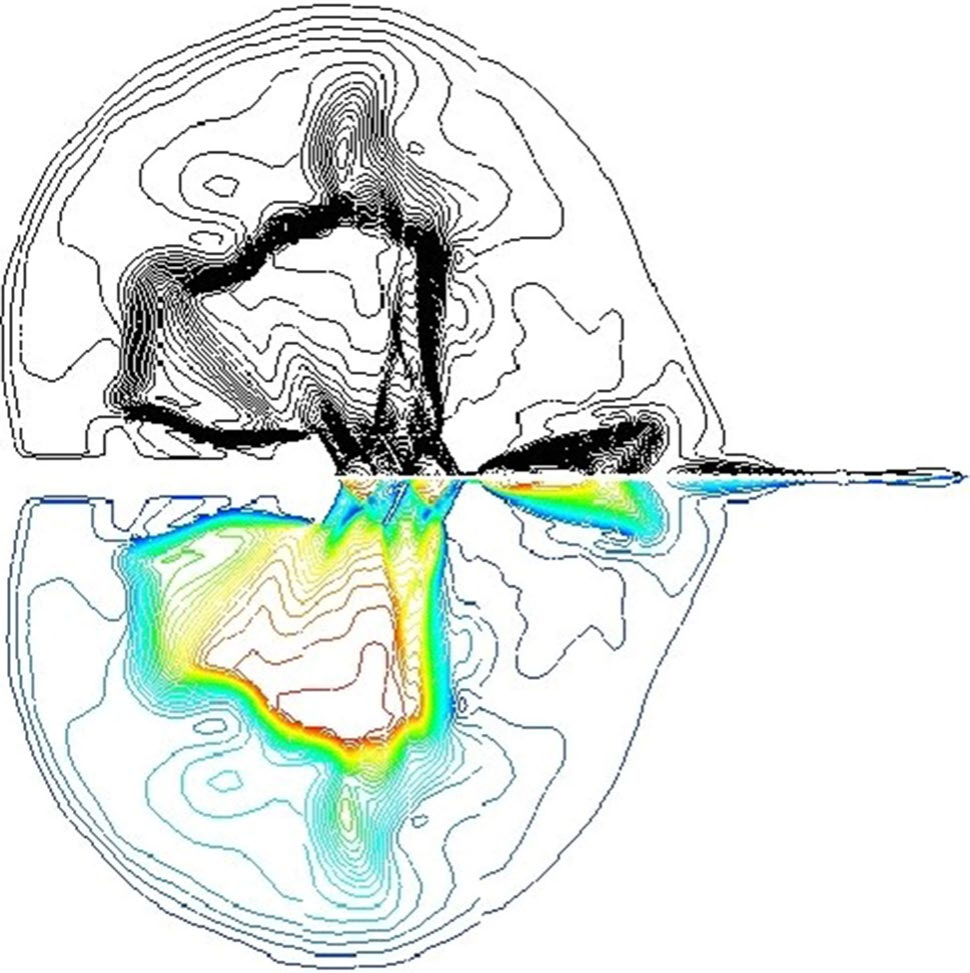
Comparison of numerical simulation and test.

**Figure 15:**
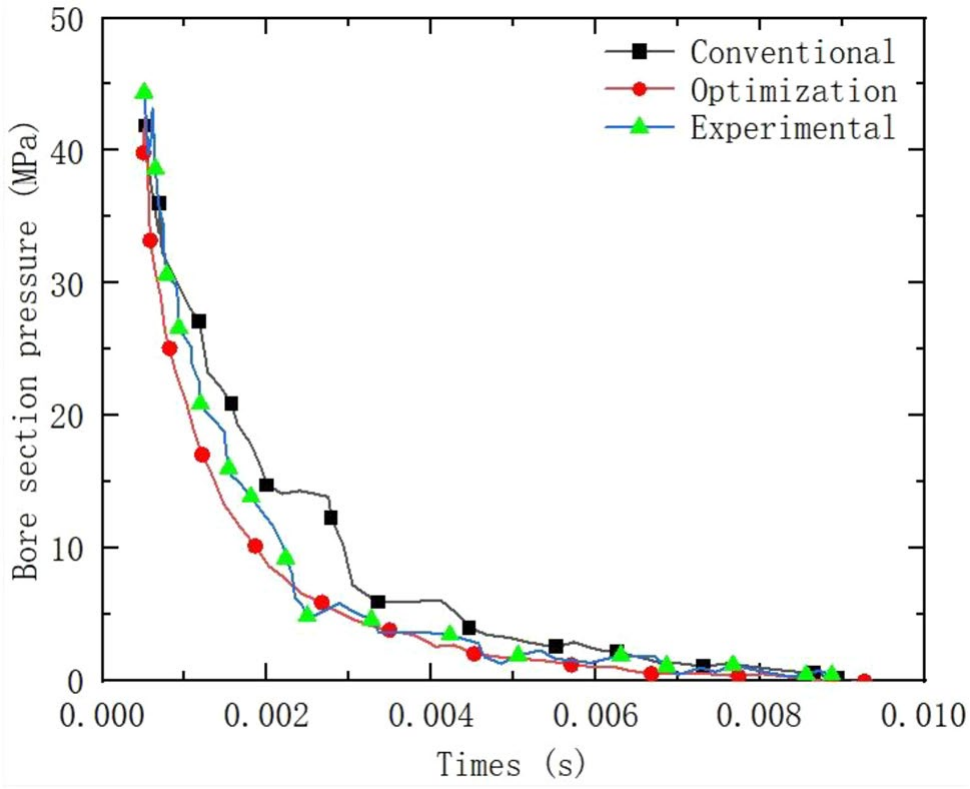
Comparison of three rifled cross-section pressures.

As shown in Fig. 14 for the comparison of test and numerical simulation, at this time in the numerical simulation of the surge structure is still the main axis, the surge area is also gradually expanding, with the forward motion of the projectile, the Mach disk is gradually changed from the disc to the vertical axis, and the surge mechanism is being shaped from the main axis into an ellipsoid shape. The gas jet from the double chamber orifice rapid jet expansion, jet tail depression, in the edge of the edge of the orifice appeared in the extreme value of the surge point. As the projectile gradually breaks through the Mach disk, the subsequent shock head will be reduced, and the shock wave structure will develop to the bowl. The test of the real situation is basically consistent. As shown in Fig. 15 for the numerical simulation and test to monitor the bowl cross-section pressure curve of the brake, as shown in the figure, the optimized bowl wall pressure is significantly reduced, and the optimized type and the traditional type of brake pressure change curve are basically consistent with the test, which can prove the accuracy of the numerical simulation.

#### Analysis of numerical simulation results

**Figure 16:**
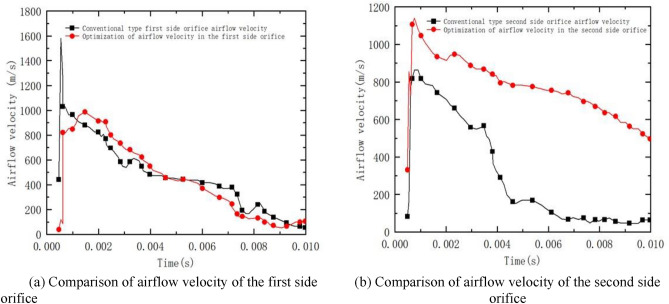
Comparison of airflow velocity of optimized one and two side orifices.

From Fig. 16a, it can be seen that the maximum velocity of the pyrotechnic gas flow in the first side orifice section of the conventional type brake is about 1600 m/s, and it rapidly decreases to about 1000 m/s in a very short time. The maximum velocity of the optimized powder gas flow in the first side aperture cross-section is about 1100 m/s, and the decreasing trend of the optimized first side aperture velocity is obvious.

From Fig. 16b, it can be seen that the velocity of the exit section of the second side orifice of the conventional type is about 810 m/s at maximum. With the decrease of gas pressure in the receding cavity, the airflow velocity of the exit section of the side aperture gradually decreases. After optimization, the exit velocity of the second side orifice is higher than that of the conventional type.

**Figure 17:**
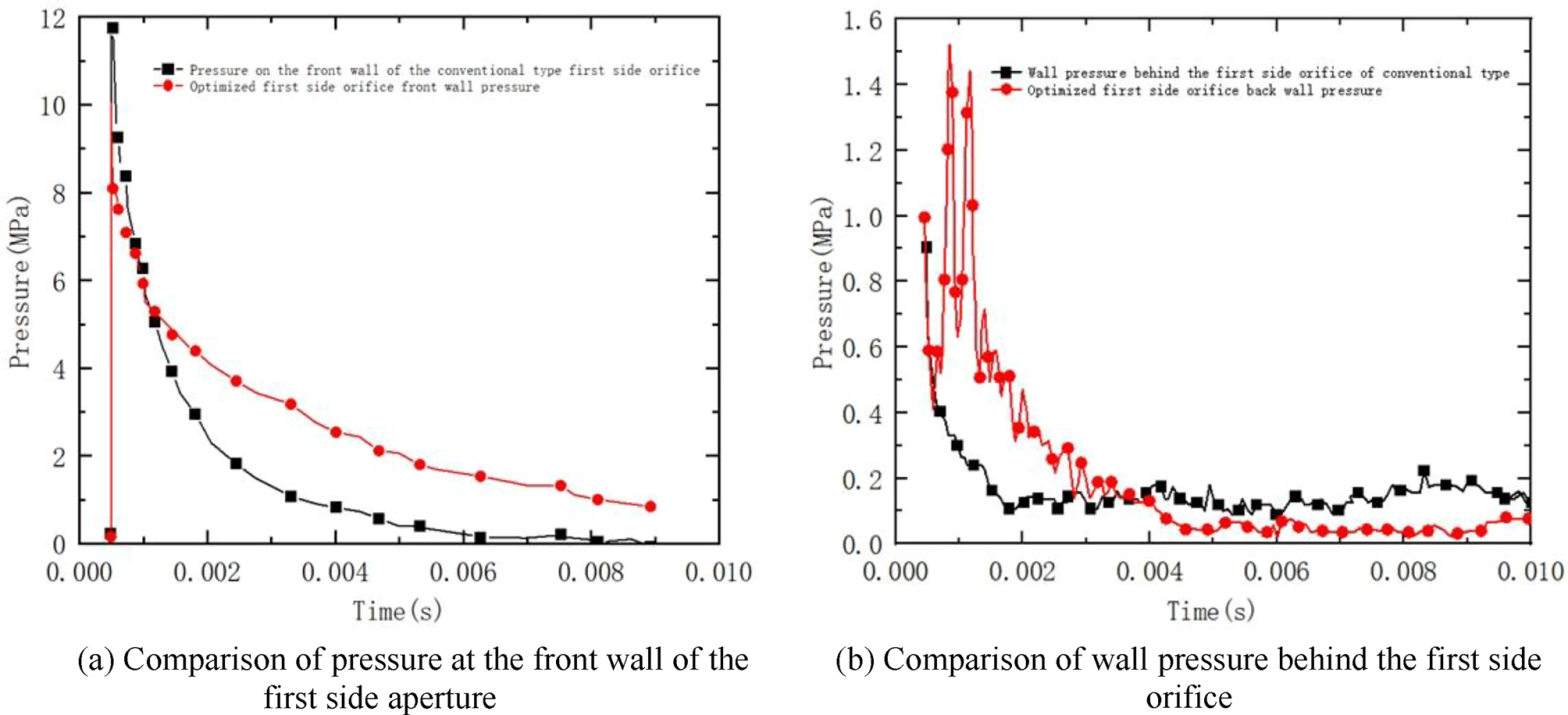
Comparison of wall pressure before optimizing one side of the orifice.

As can be seen from Fig. 17a, after the projectile exits the chamber, the powder gas in the chamber is ejected out of the chamber cross-section and enters the first receding cavity of the brake to expand, and then ejected from the first side orifice. In this process, the maximum pressure at the front wall of the first side orifice of the conventional type is about 12 MPa, and the maximum pressure of the optimized type is about 8 MPa, and the trend of the front wall decreases obviously after optimization. From Fig. 17b the wall pressure on the back wall of the first side orifice is subjected to a larger wall pressure fluctuation and force compared to the pre-optimization, the pressure on the back wall of the side orifice is elevated.

**Figure 18:**
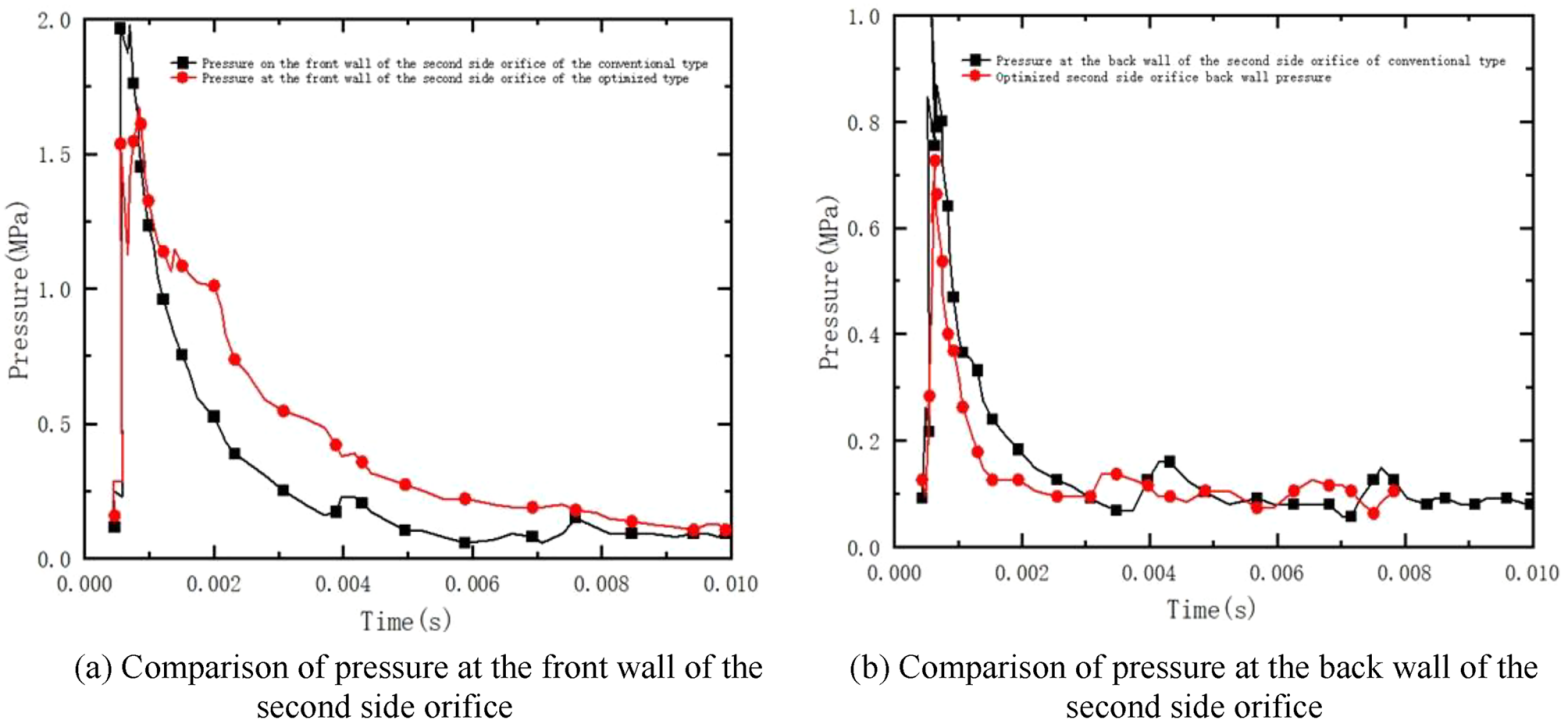
Comparison of wall pressure after optimizing the two-sided orifice.

As shown in Fig. 18a, the comparative pressure curves of the front and rear wall surfaces of the second side aperture are shown. From the curve, it can be seen that the gunpowder gas in the chamber is ejected out of the rifling section into the first receding chamber of the brake to expand, and part of it is ejected from the first side aperture and part of it enters the second receding chamber to continue to expand. In this process, the maximum pressure at the front wall of the second side orifice before optimization is about 2.0 MPa, and the maximum pressure at the front wall of the second side orifice after optimization drops to 1.7 MPa. The overall decreasing trend is smooth and similar, and it drops to a low point and stabilizes within 10 ms. From Fig. 18b, it can be seen that the maximum wall pressure before and after optimization is about 1.0 MPa. After optimization, the maximum pressure at the back wall of the brake is reduced to 0.7 MPa, and the overall decreasing trend is similar to that at the front wall, which gradually decreases to a low point and remains stable.

## Experimental analysis

### Experimental protocol and setup

**Figure 19:**
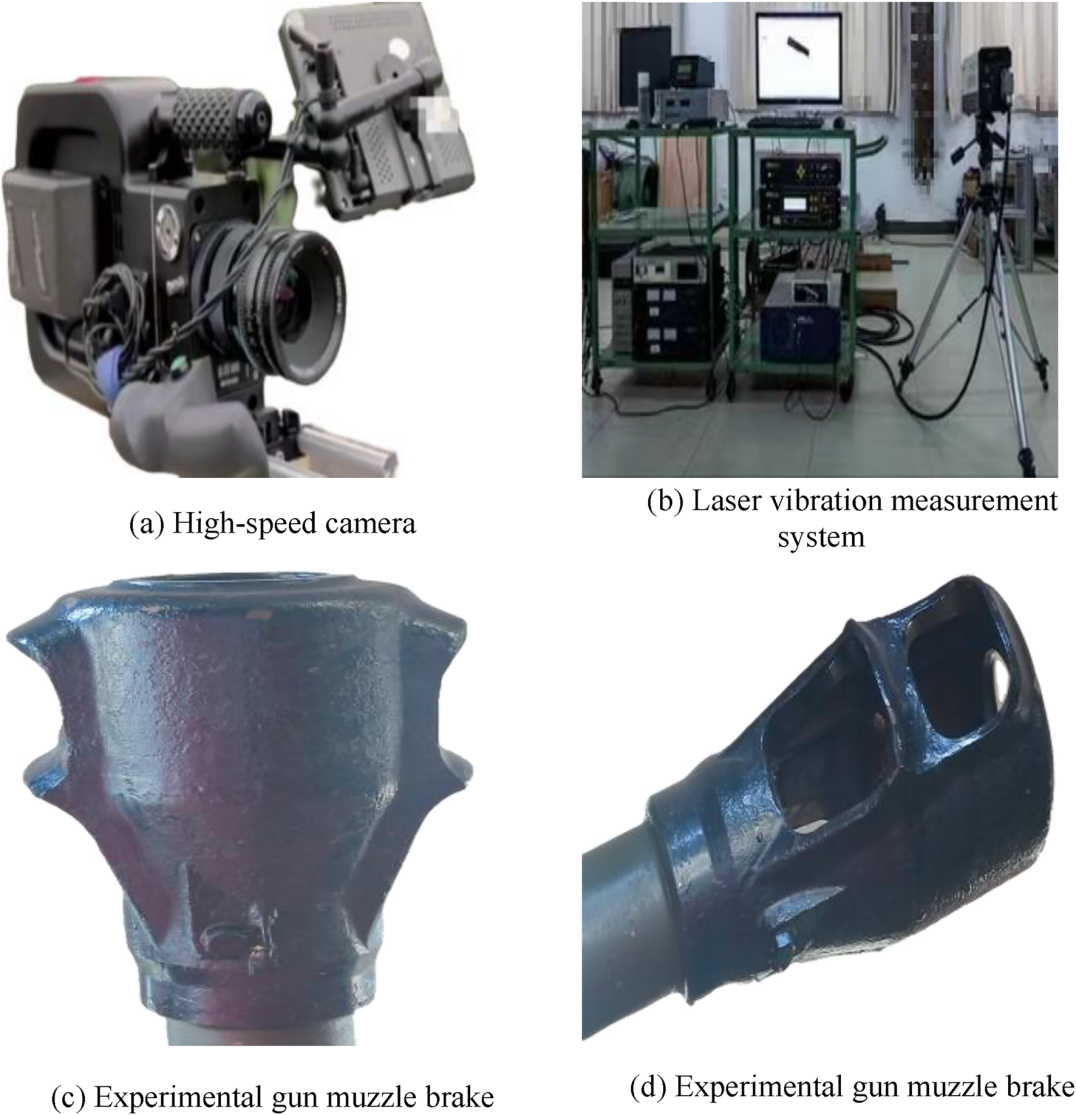
Part of the displayable experimental apparatus.

The experimental apparatus available to the public for this shooting experiment is shown in Fig. 19. In this shooting experiment, a total of 8 rounds were fired in two states, and four rounds were fired from each of the conventional and optimized brakes.

A certain type of cannon was used in this shooting, with a maximum range of 15650 m.

The maximum range was 15,650 m, with a high and low firing angle of -7° to + 35°, an average firing rate of 15–20 rounds/min, a full marching weight of 1725 kg, a 54° directional firing range, and a gun crew of 8.

**Figure 20:**
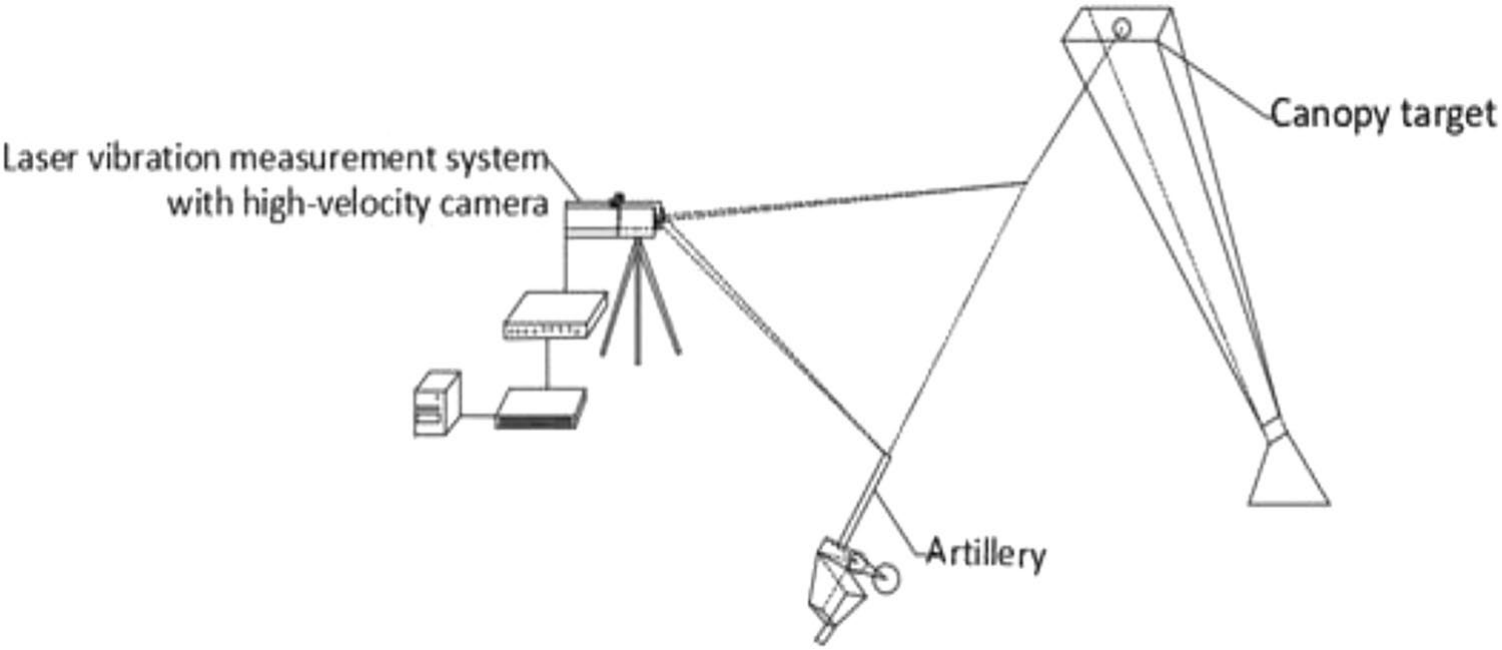
Schematic diagram of the experimental principle.

A schematic diagram of this shooting experiment is shown in Fig. 20. The weather conditions were clear, with good visibility, and the gun mount was a special fixed mount. The experimental equipment was a high-speed camera, canopy target, and laser vibration measurement system.

The experimental method was to clamp the gun launcher on a special fixed stand and use a pull cord to fire. The laser vibrometer was used to simultaneously measure the recoil speed, initial velocity, and fall speed of the recoil body. High-speed photography of the airflow and body tube movement was also conducted.

Table 2 shows the initial velocity of the projectile, the recoil velocity at the exit of the projectile, and the recoil velocity at the end of the recoil period measured by the laser vibrometer for each of the four rounds of the optimized and conventional models. The difference rate was 3.56%, while the conventional type had the lowest initial velocity of 780.80 m/s and the highest velocity of 807.00 m/s. The minimum velocity of the conventional type projectile exiting the chamber was 778.90 m/s and the maximum was 795.90 m/s, with a difference rate of 2.18%. The highest recoil velocity of the optimized recoil body at the projectile exit was 6.35 m/s and the lowest was 6.12 m/s, with a difference rate of 4.4%. Compared with the conventional recoil body, the minimum recoil velocity at the projectile exit was 6.57 m/s and the maximum recoil velocity was 6.72 m/s, with a difference rate of 2.13%, which was an increase of 2.27%. The minimum recoil velocity of the optimized recoil body at the end of the after-effect period was 4.07 m/s and the maximum was 4.33 m/s, which was 1.05 m/s and 0.99 m/s lower than that of the conventional type. 25.80% and 22.86% lower respectively, with a significant reduction effect.

**Table 2:** Recoil body recoil velocity and projectile initial velocity test results.

Programs	Muzzle velocity of projectile *v*_*o*_ (m/s)	The recoil velocity of the projectile when it exits the muzzle *v*_1_ (m/s)	Recoil velocity at the end of the recoil body after-effect period *v*_*z*_ (m/s)
Optimized type	780.80	6.12	4.12
	783.80	6.17	4.11
	777.00	6.10	4.07
	807.00	6.35	4.33
Traditional type	778.90	6.57	5.12
	778.30	6.55	5.15
	781.70	6.58	5.15
	795.90	6.72	5.32

### Results and analysis

**Figure 21:**
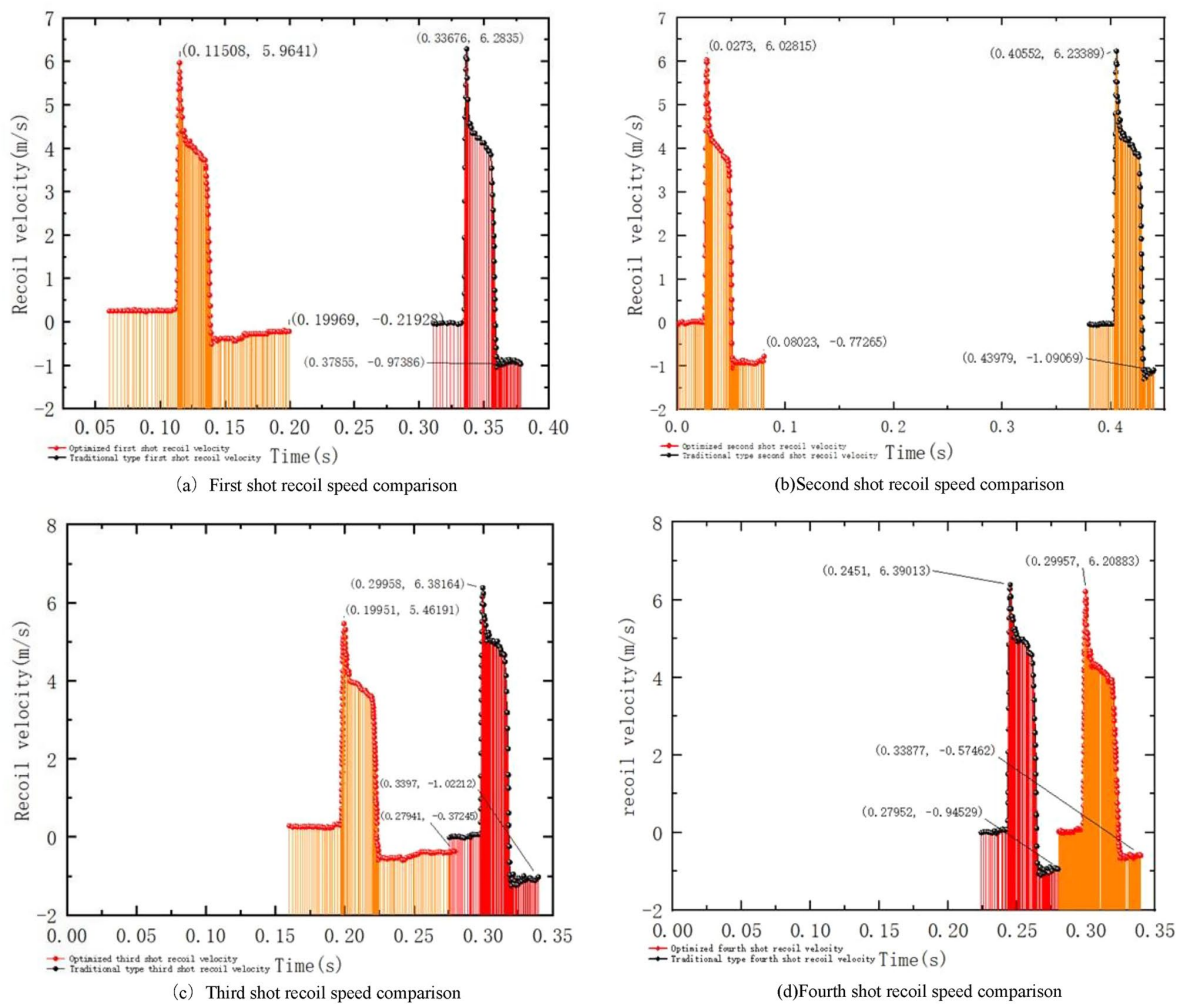
optimization type and traditional type shooting experimental recoil body speed comparison.

As Fig. 21 shows the comparison of the recoil velocity between the optimized and conventional firing experiments. After monitoring the recoil velocity of the gun frame assembly, barrel assembly, and puller handle, the peak recoil velocity of the first round with the optimized brakes is 0.31 m/s lower, the second round is 0.21 m/s lower, the third round is 0.91 m/s lower, the fourth round is 0.18 m/s lower, and the average is 0.40 m/s lower than that of the conventional brakes. The recoil speed extreme value difference decreased by 0.44 m/s for the first shot, 0.11 m/s for the second shot, 0.27 m/s for the third shot, 0.19 m/s for the fourth shot, and 0.25 m/s for the average.

**Figure 22:**
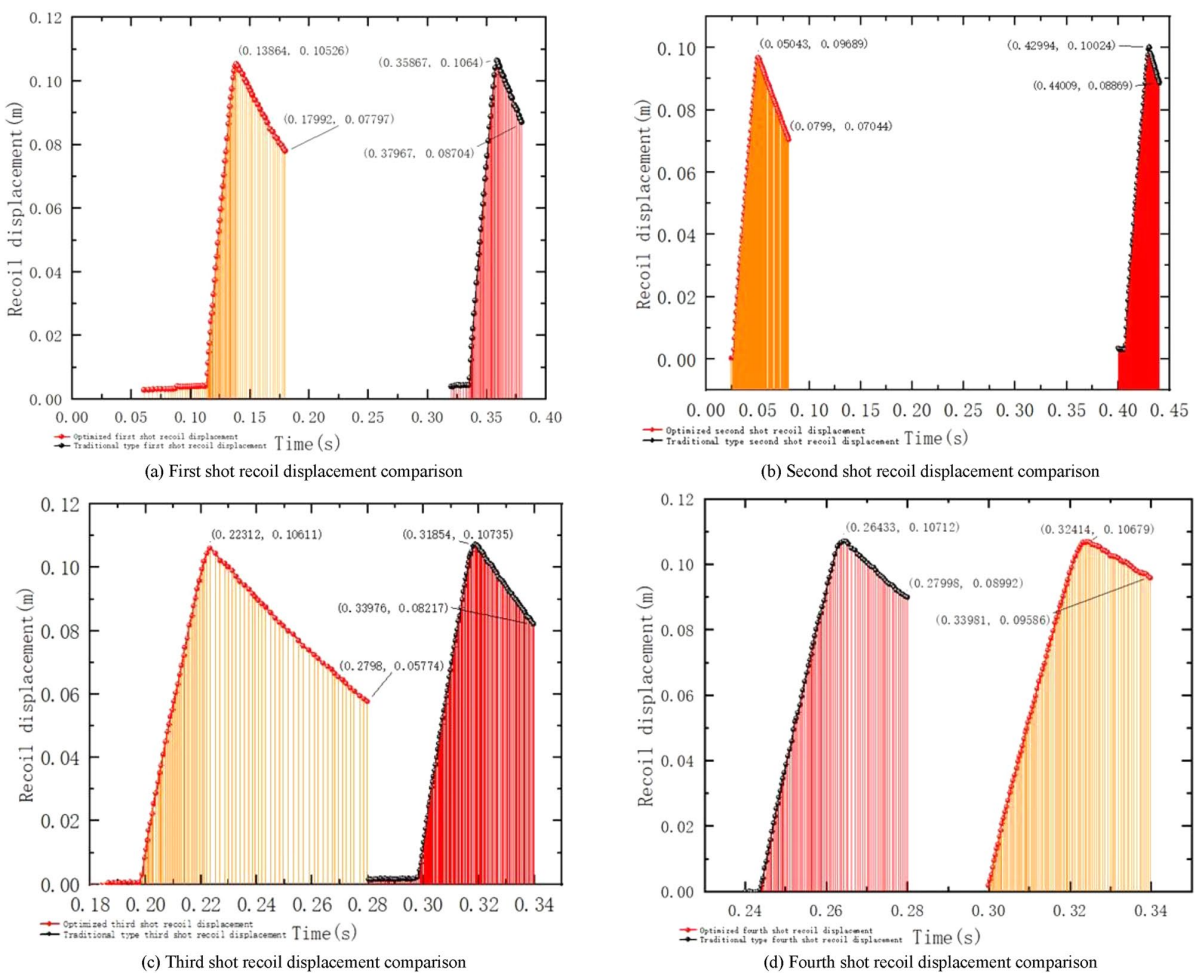
optimization type and traditional type shooting experiment recoil body displacement comparison.

Figure 22 shows the comparison of the recoil displacement between the optimized type and the conventional type of shooting experiment for this shooting experiment. After monitoring the recoil velocity of the gun frame assembly, barrel assembly, and puller handle, the peak recoil velocity with the optimized brakes was reduced by 0.00114 m for the first shot, 0.00335 m for the second shot, 0.00124 m for the third shot and 0.00033 m for the fourth shot, with an average reduction of 0.001502 m.

From the momentum theorem, the free recoil body momentum during the internal ballistic period is obtained as:

12 $$m_{H} v_{HK} = mv_{0} + 0.5m_{\omega } v_{0}$$

The free recoil impulse during the after-effect period is:

13 $$I_{HM} = \int_{0}^{{t_{1} }} {F_{pt,T} {\text{d}}t}$$

Then the total momentum of the recoil body free recoil is:

14 $$I_{H} = m_{H} v_{H} + I_{HM} = mv_{0} + 0.5m_{\omega } v_{0} + \int_{0}^{{t_{1} }} {F_{pt,T} {\text{d}}t}$$

There are two methods of calculating the efficiency of the braking brake: the energy method and the impulse method. For small-caliber weapons, the influence of the braking brake quality factor is greater, and the impulse method is mostly used for calculation. The momentum method is used to calculate the efficiency of the brake as follows

15 $$\eta = \frac{{m_{H} v_{H\max } - m_{HZ} v_{HZ\max } }}{{m_{H} v_{H\max } }} = 1 - \frac{{m_{HZ} v_{HZ\max } }}{{m_{H} v_{H\max } }}$$

In this paper, the momentum method is used to calculate the efficiency of the brake for each scheme.

Calculate the decommissioning efficiency of the conventional type decommissioner as:

$$\eta_{z} F_{1} = \left( {1 - \frac{{Q_{z} V_{z} }}{{Q_{h} V_{h} }}} \right)\% = \left( {1 - \frac{12.8 \times 5.14}{{12.2 \times 9.18}}} \right)\% = 41.3\%$$

Calculate the decommissioning efficiency of the optimized decommissioner as:

$$\eta_{z} F_{1} = \left( {1 - \frac{{Q_{z} V_{z} }}{{Q_{h} V_{h} }}} \right)\% = \left( {1 - \frac{13.8 \times 4.10}{{12.2 \times 9.18}}} \right)\% = 49.5\%$$

**Figure 23:**
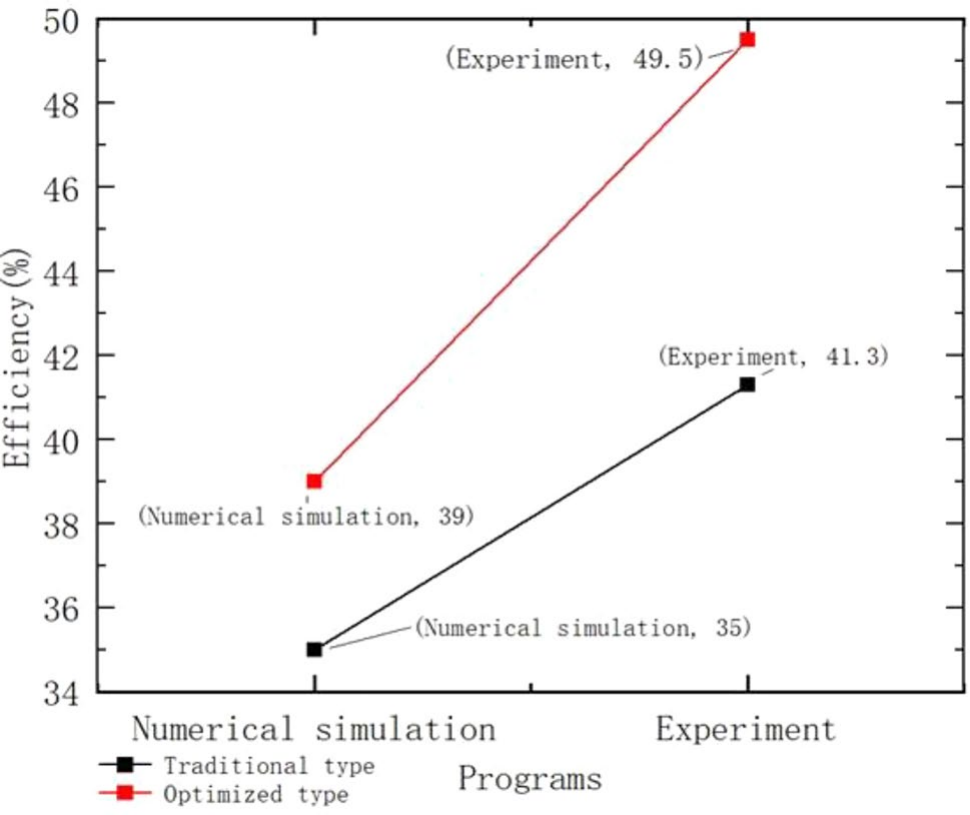
Efficiency comparison.

According to Table 3 above: With the same projectile mass of 0.098 kg and the same charge, the recoil momentum and the total momentum of the free recoil body in the inner ballistic period were both kept the same by the numerical simulation with the fluid calculation software. After optimization, the maximum velocity with recoiler was reduced by 0.35 m/s compared with that before optimization, and the recoil impulse with recoiler was reduced by 5.27kgm/s during the after-effects period, which was calculated to be 39% of the recoil efficiency of the optimized type by numerical simulation, which was 4% higher than that of the conventional type. As shown in Fig. 23, Compared with the measured value of 49.5%, the error value is 10.5%. The error value is 6.3% compared to the measured value.

**Table 3:** Comparison of efficiency calculations for brakes.

Parameters	Traditional type	Optimized type
Projectile quality (kg)	0.098	0.098
Charge quantity (kg)	0.032	0.032
Recoil body mass (kg)	15.00	15.00
Non-rifled after-effect period powder gas impulse (kgm/s)	25.30	25.30
Recoil momentum at the end of internal ballistics (kgm/s)	94.62	94.62
Total momentum of sitting body after free recoil (kgm/s)	119.92	119.92
Maximum recoil velocity of light bore port (m/s)	7.99	7.99
Maximum recoil speed with brake (m/s)	5.23	4.88
With brake after-effect period after sitting impulse volume(kgm/s)	-16.20	-21.47
With brake after sitting body impulse(kgm/s)	78.42	73.15
Numerical simulation efficiency (%)	35.00	39.00
Experimental efficiency (%)	41.30	49.50

## Conclusion

In this paper, the air velocity and cross-sectional pressure of the first and second side orifices of the double-cavity brake were monitored and plotted numerically. After the shape optimization, the same numerical simulations were performed and compared with the pre-optimization one by one. After that, the recoil generator before and after optimization was subjected to a four-round firing experiment using a high-speed camera and a laser vibrometer. The recoil velocity and displacement obtained from the experiments were plotted separately for visual comparison, and the efficiency was finally calculated by the momentum method. The following conclusions were obtained:

1. The optimized side aperture exit cross-section pressure is significantly lower than the optimized exit cross-section pressure, and the reduction of wall pressure after the improvement indicates that the optimized recoil-making efficiency is effectively improved.
2. The optimized type of recession maker makes the recoil impulse of the recession maker effectively reduced compared with that before the improvement, and the recession maker efficiency is significantly better than that of the conventional double-cavity recession maker.
3. The pressure of the first side aperture of the double-chamber brake is much greater than that of the second side aperture, and the impact on the first side aperture is significantly greater during the firing process.
4. The recoil velocity and displacement obtained from the experimental apparatus under good meteorological conditions, four rounds of firing experiments were conducted on each of the guns equipped with the conventional and optimized type of recoilers, and it is obvious that the optimized type of recoilers reduces the recoil displacement and the recoil velocity significantly, which effectively reduces the recoil impulse.
5. The difference between the numerical simulation efficiency and the experimental efficiency of the conventional type recoil brake is 6.3%, while the difference between the numerical simulation efficiency and the experimental efficiency of the optimized type recoil brake is 10.5%, which is generally in line with the expected assumption.
6. After the gun is fitted with the double chamber brake, the maximum recoil velocity is effectively reduced, and the resulting recoil impulse and overall recoil impulse. The forward impulse generated by the recoiling body provides a restraining force on the gun body, so that the recoil impulse generated by the combined force of the gun bore is reduced, thus effectively reducing the firing load and gun recoil kinetic energy acting on the gun frame.
7. Through the use of experimental equipment in the case of good meteorological conditions, assembled with the traditional and optimized brake in each of the four rounds of shooting experiments, the recoil velocity and displacement can be intuitively seen in the optimized brake so that the recoil body displacement is reduced, the recoil velocity is significantly reduced, and consequently effectively reduce the recoil impulse.

### Research Implications and future perspectives

In this study, numerical simulations and experiments of a double-chambered brake were conducted based on fluid computational mechanics. At present, there are fewer domestic and foreign studies on the flow parameters and optimization scheme of the double-chamber brake, and the ones that can be tested are even more valuable. In this paper, the use of computational fluid dynamics in the dynamic grid technology research, all grids using structural mesh, the efficiency and accuracy of the entire numerical simulation has a greater significance. The test shot after the numerical simulation has a positive significance on the design and optimization of the subsequent brake ‘’structure. And this study follows a more scientific and rigorous program in the writing process, the research method can provide a reference for the subsequent research on the retreating brake.

The purpose of this study is to numerically simulate and test a double-chamber muzzle brake based on fluid computational mechanics. However, because the numerical simulation does not allow for a complete reduction of the model during meshing, the model is partially simplified, which can lead to an idealization of a portion of the data from the numerical simulation. Because of the difficulty of the tests, only eight shots were fired in this study, and it is hoped that in future research, more shots can be fired under more ideal conditions to make the test results more convincing. It is also hoped that future work will be directed toward the multi-component combustion of gunpowder and fluid–solid coupling, which can make the research more cutting-edge and practical.

## Data Availability

All data included in this study are available from the corresponding author upon request. The preprint on the web is a returned manuscript, the journal director has been contacted to withdraw the web preprint, and there is no copyright dispute over the paper.
